# Scaling up tuberculosis case finding via private providers in Ghana: an impact evaluation using interrupted time series

**DOI:** 10.3389/fpubh.2025.1598269

**Published:** 2025-08-26

**Authors:** Kenneth Mawuta Hayibor, Ernest Kenu, Gloria Ivy Mensah, Dziedzorm Awalime, Jabina Anaman, Adwoa Asante-Poku, Olena Ivanova, Bakuli Abhishek, Andrea Rachow, Nortey Nii Hanson-Nortey

**Affiliations:** ^1^Center for International Health, Ludwig-Maximilians-Universität, Munich, Germany; ^2^Noguchi Memorial Institute for Medical Research, University of Ghana, Accra, Ghana; ^3^School of Public Health, University of Ghana, Accra, Ghana; ^4^Aurum Institute Ghana, Accra, Ghana; ^5^Institute of Infectious Diseases and Tropical Medicine, LMU University Hospital, LMU Munich, Munich, Germany; ^6^German Centre for Infection Research (DZIF), Partner Site Munich, Munich, Germany; ^7^Unit of Global Health, Helmholtz Zentrum München, German Research Centre for Environmental Health (HMGU), Neuherberg, Germany

**Keywords:** private healthcare providers, public-private mix, tuberculosis, active case notification, community engagement

## Abstract

**Background:**

Although TB services are free in Ghana, TB case detection remains low and mostly limited to public facilities. To address this, a Public-Private Mix (PPM) Directly Observed Therapy (DOT) model was introduced, involving community private healthcare providers and the National Health Insurance Scheme (NHIS) to boost TB case detection rates.

**Methods:**

This impact evaluation focuses on four key interventions targeting vulnerable populations in Ghana’s two largest metropolitan areas between the last quarter of 2018 and the first quarter of 2020. Screening and TB register data were collected from implementing facilities, along with TB case notifications from 2015 to 2022 for both intervention and control areas. Comparative interrupted time series (ITS) analysis was used to evaluate the effect of the interventions on quarterly TB case notifications.

**Results:**

During the intervention period, a total of 563,868 persons were screened for TB, 12,121 of these were presumptive for TB and 590 persons were diagnosed with TB. Of the diagnosed TB cases, 95.3% (562) were bacteriologically confirmed. The overall TB screening yield was 104.6 cases per 100,000 population. In the intervention area, TB case notifications increased from 1,392 cases in 2018 to 1,462 cases in 2019 while they decreased from 853 to 778 in the control area. The ITS analyses detected positive post-intervention trend differences in all forms of TB and bacteriologically confirmed TB notification case rates between the intervention and control areas.

**Conclusion:**

Expanding free TB services through a PPM DOT model and sustained community engagement can increase TB case detection in urban areas. National TB programs should adopt and scale this approach to enhance TB surveillance and control.

## Introduction

Despite significant progress in controlling and preventing tuberculosis (TB), the disease remains a global health threat (1). A major barrier to an adequate TB response is the fact that millions of people with TB are still missed by the current routine health systems and national TB control programs (2). Of the estimated 10.6 million people who fell ill with TB worldwide in 2021, 4.2 million were not diagnosed nor officially reported to national authorities (1). Furthermore, after the start of the COVID-19 pandemic in 2020, TB case notifications in 2021 were still 10% below the level of 2019 (2).

For TB control efforts to be successful and achieve the 2030 END TB targets, it is important to ensure universal access to quality TB services for all (3). A major challenge to achieving this is the lack of systematic engagement of all healthcare providers, especially those in the private sector (4, 5). Public-Private Mix (PPM) and community engagement have been demonstrated to be very effective in increasing case finding in high-burden countries (6). Where PPM is well implemented, it has led to increased TB case finding and successful treatment outcomes (7, 8).

TB services are free and available in public facilities in Ghana, however, the TB case detection rate remains below the expected level. TB prevalence in Ghana currently stands at 280 per 100,000 population (9). Of the total estimated incident TB cases in Ghana from 2015 to 2020, only 29–34% were detected and notified to the national tuberculosis programme (NTP) (10).

PPM is a critical component of Ghana’s National TB Strategic Plan (9). The private sector in Ghana is increasing, and the number of patients who demand care in private health facilities is increasing as well. According to the Ministry of Health’s Holistic assessment of the health sector program of work in 2018, the private sector contributed 21% of Ghana’s total out-patients department (OPD) attendance in 2017 (11).

Targeted TB case finding using private health care providers in urban slums in Accra was implemented successfully but not scaled up due to lack of sustainable funding (9). At the peak of PPM implementation in 2009, the private health sector contributed 11% of total TB case notifications in the metros; however, this reduced significantly due to limited support from the NTP. In 2017, the two cities contributed 9.8% (of which the private sector contribution was almost absent) of national TB case notification despite accounting for 14% of the national population (12). This demonstrated an opportunity to invest in increased TB notification through targeted case finding via private healthcare providers through a PPM Directly Observed Therapy (DOT) model. In this PPM DOT model, private healthcare providers screened clients and community members for TB and the National Health Insurance Scheme (NHIS) reimbursed the cost of enrolled patients’ consultation and case review in private health providers’ facilities as an incentive. This improved model, unlike the previous model, also actively engaged private pharmacies and community health volunteers in its implementation. This study aimed to assess whether a multifaceted PPM DOT model intervention involving private healthcare providers, pharmacies, community health volunteers, and NHIS reimbursement can significantly improve TB case detection among vulnerable populations in Ghana’s two largest metropolitan areas.

## Methodology

### Study design

We employed a prospective impact evaluation approach to assess multifaceted active-TB case-finding interventions among vulnerable persons living in urban slum communities.

### Setting

In 2021, the population of Ghana was 30.8 million with 58% of the population living in urban areas (13). More than one-third of the population lives in the Greater Accra Region (capital: Accra) or Ashanti Region (capital: Kumasi), which are among the regions with the highest burden of TB in the country.

The intervention was implemented in selected sub-metropolitan areas within the Greater Accra and Ashanti Regions. These areas included Ablekuma, Ayawaso, Ashiedu-Keteke, Korley-Klottey, and Okaikoi in Greater Accra, as well as Asokwa, Bantama, Manhyia South, and Manhyia North in Ashanti. The selection of these sub-metros was based on three main criteria: a high burden of TB with low case detection rates (based on NTP data (9)); urban slum characteristics with high population density; and the availability of private health facilities capable of providing PPM DOT TB services.

**Figure 1:**
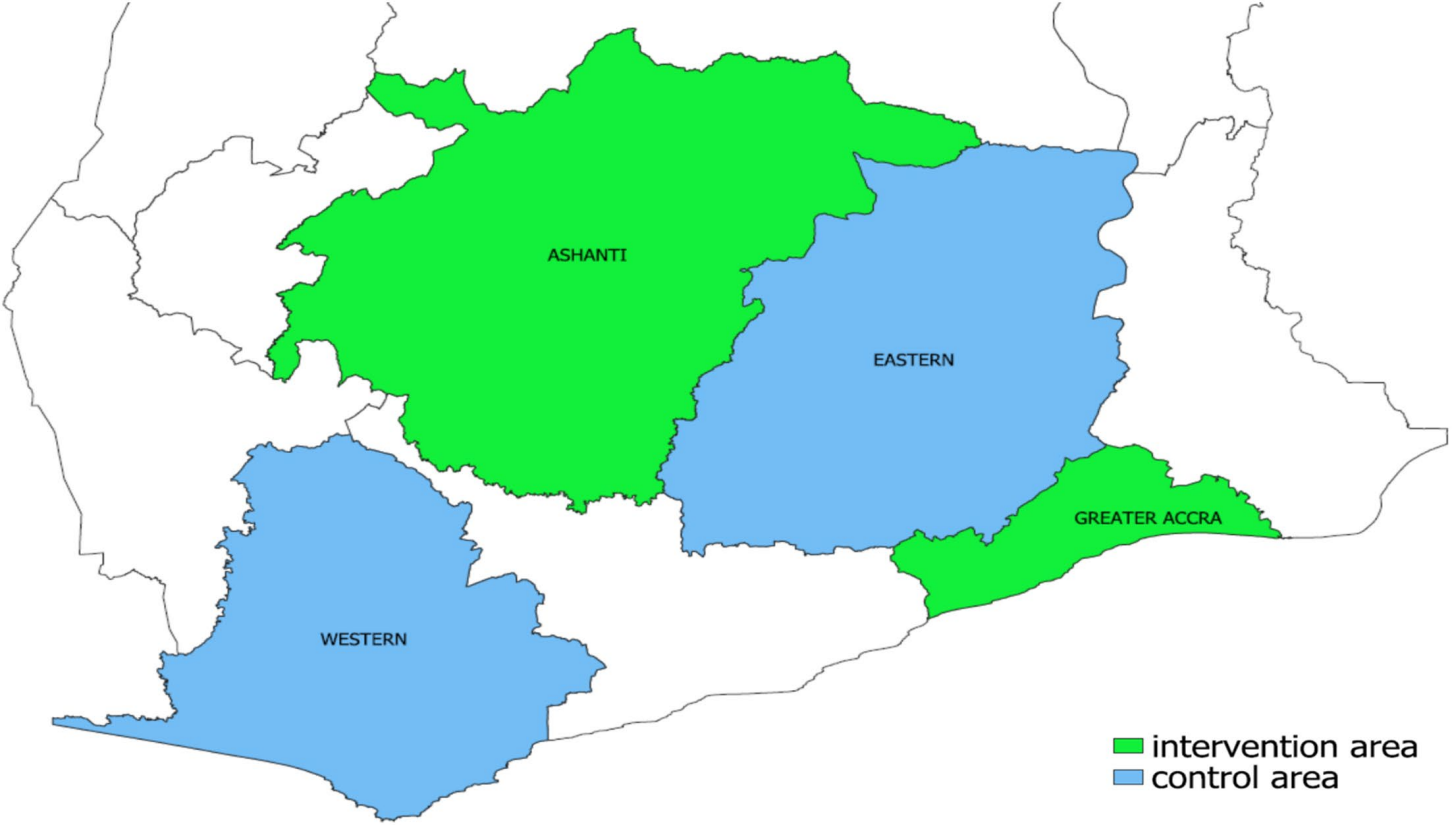
Regional map of Ghana showing the intervention area (Greater Accra and Ashanti regions) and control area (Eastern and Western regions).

To assess the effects of the intervention, sub-metros from the Western and Eastern regions were selected as control areas. The control sub-metros included Ahanta West, Sekondi-Takoradi, Shama, and Jomoro in the Western Region; and Suhum Municipal, Nsawam Adoagyiri, New Juaben, and Yilo Krobo in the Eastern Region. These were selected through a process using baseline criteria similar to the intervention areas. Additionally, none of the control sub-metros had participated in PPM TB programs during the study period, reducing the risk of contamination. Figure 1 shows the regions included in the study.

### Interventions

Four active TB case-finding interventions were implemented by a consortium led by the Aurum Institute Ghana and two other partners, the TB Voice Network (TBVN) and the Alliance for Global Action (AGA) with collaboration from the NTP. The interventions included intensified case finding in private health facilities, intensified case finding in community pharmacies and over-the-counter medicine sellers (OTCMS), community active case finding in urban slums and contact tracing among household contacts of index TB cases. The project started with training in October 2018 and ended in March 2020. There were no similar interventions before the start of these interventions in either the intervention or control areas.

#### Intensified case finding (ICF) in private health facilities

Seventy-two private healthcare clinics were selected for the implementation of project activities. All consenting clients who visited these facilities were screened symptomatically for TB. All persons presumed to be positive for TB sputum samples were collected for testing and a few of the persons were referred to do a chest X-ray. Persons found positive were enrolled in treatment in private or public facilities according to proximity. To enhance laboratory confirmation of TB, four new GeneXpert machines were installed across four facilities as part of the project’s strategy.

#### Intensified case finding in community pharmacies and OTCMS

Sixty-nine pharmacies and OTCMS medicines shop attendants were recruited and trained on TB case finding and management in Accra and Kumasi (43 in Accra, 26 in Kumasi). Pharmacies and OTCMS attendants conducted symptomatic screening of consenting clients who visited these shops. Persons presumed positive for TB were referred to designated private health facilities through assigned trained TB detectors (TBDs) for diagnosis. Persons found positive were enrolled for treatment in either private or public facilities, according to proximity.

#### Community active TB case finding in urban slums

Community active TB case search activities and events were implemented in urban slum communities within the project’s sub-metros by trained community TB Detectors (TBDs) and health personnel who organized various community engagement and TB awareness events. On each of the events, all consenting participants were symptomatically screened for TB, a digital chest X-ray and sputum samples of presumptive TB persons were tested for TB. Those who tested positive for TB were subsequently connected to initiate free treatment at either private or public facilities, depending on their preference.

#### Contact tracing among index TB cases

Contract tracing was conducted among newly diagnosed TB patients. A TBD was assigned to each newly diagnosed TB patient and all members of the person’s household and workplace, if available, were contacted, screened, and their samples collected and tested for TB. Those who tested positive for TB were subsequently connected to care at either private or public facilities to receive free treatment, depending on their preference.

### Data sources

Data on the number of people screened, referred, tested, diagnosed, and treated for TB during each intervention were collected using a combination of paper forms and TB registers. This data encompassed only the implementing facilities, not all facilities in the intervention area. The NTP provided quarterly TB case notification data for all facilities in the intervention and control areas. This data is aggregated and not broken down into public and private sector contributions. The aggregated data collected was from the first quarter of 2015 to the last quarter of 2022. Population data for calculating TB case notification rates was sourced from the Ghana Statistical Service, using estimates from the 2021 population and housing census for the analysis (13).

## Statistical analysis

Statistical analyses were performed using R Statistics version 4.2.2. The study’s main outcome was the interventions’ yield, which was determined by the number of TB cases detected and notified during the intervention period. Quarterly TB case notification rates were calculated for pre-intervention (2015Q1-2018Q3), intervention (2018Q4 - 2020Q1), and post-intervention (2020Q2-2022Q4) periods taking into account the population of the intervention and control areas. To assess the impact of the intervention, we conducted an comparative interrupted time-series (ITS) analysis of aggregated quarterly TB case notification rates. The ITS analysis was based on linear regression model for the quarterly rates of incidence observed with a separation into control phase and intervention implementation phase. Potential confounders were addressed by selecting control areas with similar demographics that did not have overlapping TB interventions, and by modeling COVID-19 disruptions as a separate, time-varying confounder. Summary statistics have been reported for the pre and post-intervention phase and how the COVID-19 pandemic overlapped with the post-intervention phase for the outcome of notification rates. The plots were produced using *ggplot*2 package in R.

## Ethics and funding

Ethical clearance was obtained from the GHS Ethics Review Committee (GHS-ERC 00s/01/19) as well as a vote from the LMU Hospital’s ethics committee in Munich, Germany (23–0484). The interventions and operational research were funded by the TB REACH initiative of the Stop TB Partnership Wave 6. The sponsor had no role in the study design or data collection. The sponsor was involved in monitoring overall project performance through quarterly reports but not involved in the analysis, decision to publish, or preparation of the manuscript.

## Results

### TB screening cascade and yield per intervention

Between October 2018 and March 2020, the four active TB case-finding interventions were implemented. A total of 563,868 persons were screened for TB symptoms. Among these, 12,121 were identified as presumptive cases of TB, and 590 individuals were diagnosed with the disease. This resulted in an overall screening yield of 104.6 cases per 100,000 people. Table 1 displays the screening cascade and yield of TB per intervention. The ICF in private health clinics intervention screened the highest number (382,537) of persons for TB, and the TB screening yield was 54.6 per 100,000 population. Out of 438 household contacts of identified TB cases screened, 10 were confirmed bacteriologically to have TB, resulting in a screening yield of 2,283 cases per 100,000 or one positive case for every 44 contacts screened. The community case-finding intervention identified the highest number of diagnosed TB cases, accounting for 59.8% (353/590) of all cases detected. In comparison, the case-finding intervention that involved pharmacies and OTCMS identified the highest proportion of presumed TB cases relative to the number screened, 20.6% (1,321/6,414). However, it resulted in the lowest percentage of confirmed TB cases, with only 1.4% (18 /1,321) testing positive despite all the presumptive TB cases being tested for TB. Almost all cases diagnosed, 95.8% (565), were linked to a TB treatment facility and initiated treatment.

**Table 1:** TB screening cascade and yield per intervention from Oct 2018 to March 2020.

Indicator/Intervention	ICF in private health clinics	Community case finding	Contact tracing	Case finding in pharmacies and OTCMS	Total
No. of people screened	382,537	17,4,479	438	6,414	563,868
No. of people with presumptive TB	6,042 (1.6%)	4,702 (2.7%)	57 (13.0%)	1,321 (20.6%)	12,121 (2.1%)
No. of people tested for TB	5,071 (83.9%)	3,718 (79.1%)	57 (100%)	1,321 (100%)	10,170 (83.9%)
No. of people with laboratory-confirmed TB	186 (3.7%)	346 (9.3%)	10 (17.5%)	18 (1.4%)	562 (5.5%)
No. of people with all forms of TB	209 (4.1%)	353 (9.5%)	10 (17.5%)	18 (1.4%)	590 (5.8%)
No. of Bac + TB patients started on treatment	177 (95.2%)	331 (95.7%)	10 (100%)	18 (100%)	536 (95.4%)
No. of All Forms TB patients started on treatment	201 (96.2%)	336 (95.2%)	10 (100%)	18 (100%)	565 (95.8%)
No. needed to screen to detect 1 TB case	1830	494	44	356	956
No. needed to test among presumptive TB cases to detect one TB case.	29	13	8	73	21
Screening yield per 100,000 population	54.6	202.3	2,283	280.6	104.6

### Trends in TB case notifications

**Figure 2:**
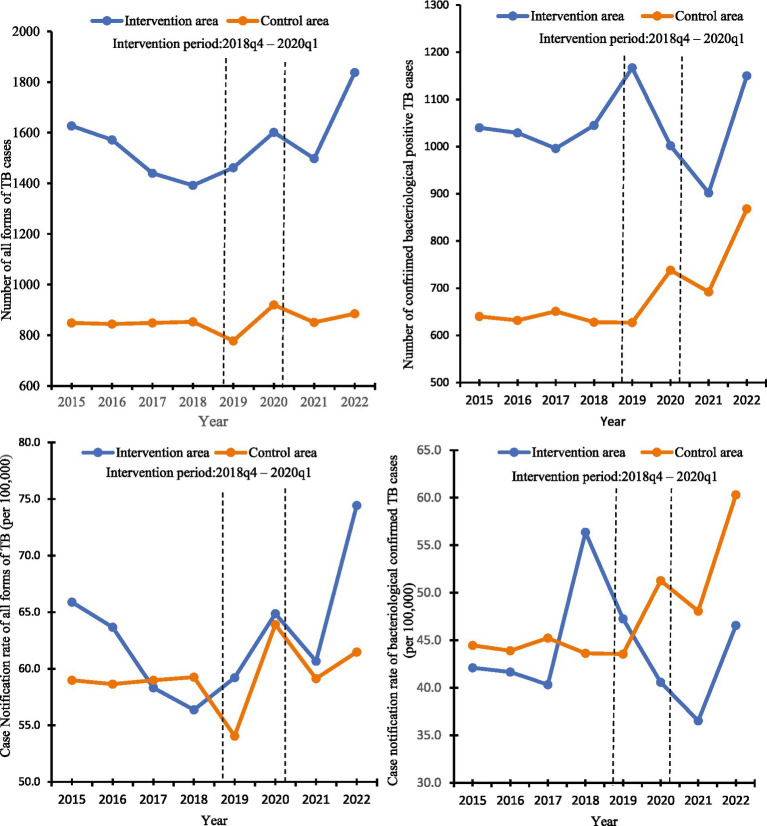
Number of TB cases detected and annual TB case notification rates in the intervention and control area, 2015 to 2022.

The annual number of TB cases and TB case notification rates (all forms and bacteriologically confirmed) detected from 2015 to 2022 in the intervention area and the control area are shown in Figure 2. There was a gradual decrease in the number of all forms of TB cases detected in the intervention area, followed by a gradual increase, just before the intervention started, which was in sharp contrast to the control area where case numbers remained relatively stable for the first 4 years, before a decline in 2019, followed by an upward trend before returning to the normal trend. Regarding the number of bacteriologically confirmed TB cases, the intervention area showed a fluctuating pattern. It reached its highest point in 2019 but experienced a significant decrease in 2021. On the other hand, the control area displayed a more gradual increase over the years, with a significant rise observed from 2020 to 2022.

The quarterly notifications for all forms of TB and bacteriologically confirmed TB cases to the NTP from the intervention and control areas from Q1 2015 to Q4 2022 are shown in Table S1 of the Supplementary material. Over the 32 quarters, in the intervention area, the average number of people with all forms of TB case notifications was 389 and the average of bacteriologically confirmed TB cases was 260; in the control area average of all forms of TB cases was 213 persons with TB the average of bacteriologically confirmed TB cases was 171 people. In the intervention area, the notification of all forms of TB cases showed variations over the years, peaking in 2021, with a substantial increase in bacteriologically confirmed TB cases. The case notification rate of all forms of TB in the intervention area was 15.7 per 100,000, while that of the control area was 14.8 per 100,000. The first case of COVID-19 was reported in Ghana in March 2020. Following this detection, the number of TB cases that were detected and notified to the NTP in the intervention area decreased from 503 in the first quarter of 2020 to 321 in the second quarter of the same year. Similarly, in the control area, the number of TB cases dropped from 252 in the first quarter of 2020 to 182 in the second quarter of the same year. This decline in TB cases continued until the second quarter of 2021.

### Interrupted time series analysis of TB notification rates

**Figure 3:**
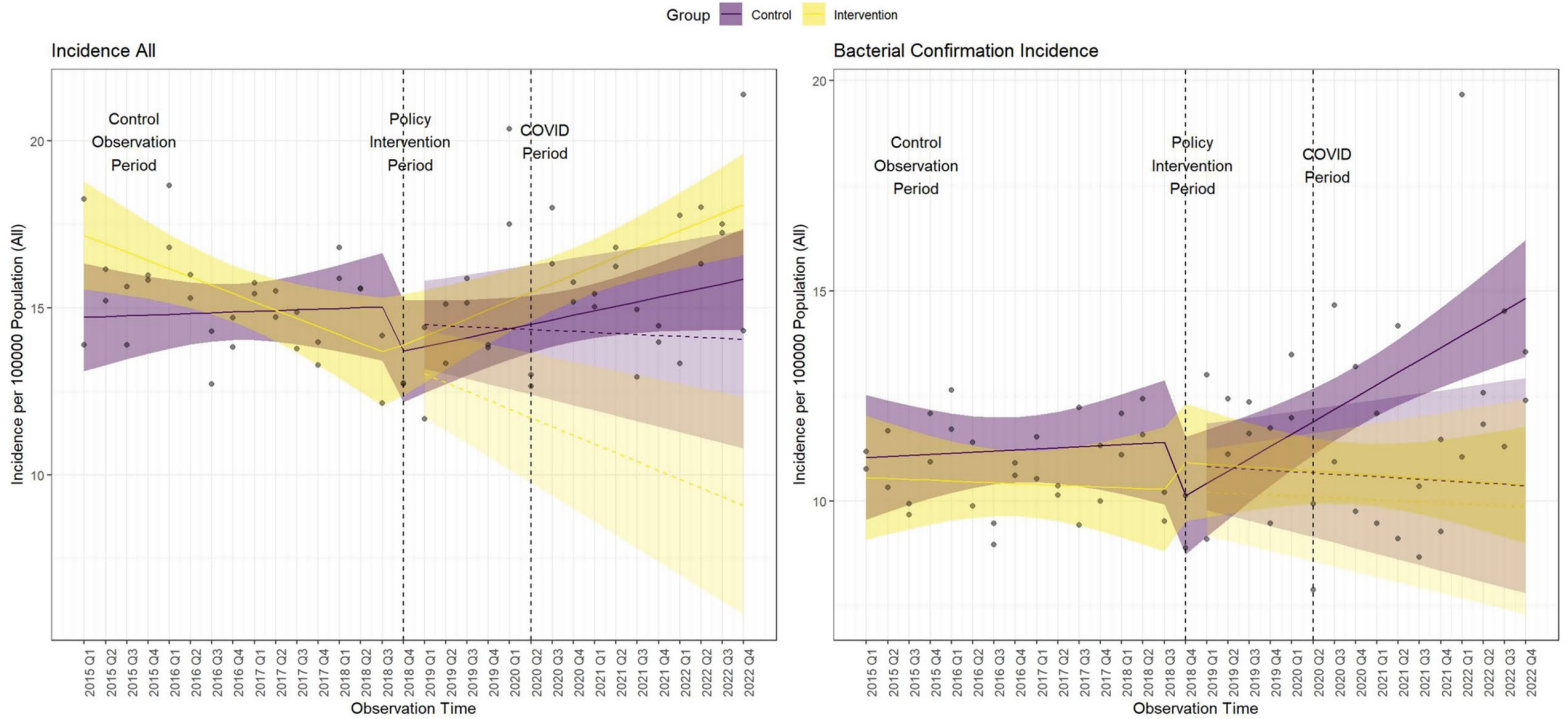
Comparative interrupted time series analysis model graphs of population-standardized quarterly tuberculosis notification rates. Dashed lines indicate the start and end of the intervention period. The COVID-19 period begins at the end of the intervention. Shaded areas represent the 95% confidence intervals (CI).

Figure 3 shows the controlled interrupted time series analysis model graphs illustrating population-standardized quarterly TB notification rates for both (1) All Forms of TB, and (2) bacteriologically confirmed TB, in the intervention and control areas. Table 2 provides a summary of the model parameters that compares these trends between the two groups. Before the intervention, the intervention area had significantly higher TB notification rates than the control area for all forms of TB cases but not for bacteriologically-confirmed TB cases [All forms of TB (*β* = 2.72; 95% CI: 0.15–5.29; *p* = 0.039), bacteriologically confirmed (*β* = −0.44; 95% CI: −2.80 – 1.92; *p* = 0.712)]. Over time, the TB case notification trend slightly increased in the control group, but the increase was only significant for bacteriologically-confirmed TB cases [All forms of TB (*β* = 0.04; 95% CI: −0.05 – 0.12; *p* = 0.393), bacteriologically confirmed (*β* = 0.09; 95% CI: −0.01 – 0.17; *p* = 0.029)]. The immediate effect of the intervention after the first quarter of its implementation showed that TB case notifications increased in the intervention area compared to the control area, although it was not statistically significant [All forms of TB (*β* = 1.39; 95% CI: −1.95 – 4.74; *p* = 0.408), bacteriologically confirmed (*β* = 2.23; 95% CI: −0.83 – 5.30; *p* = 0.150)]. Post-intervention trend, TB case notification rates significantly increased in the intervention group compared to the control for all forms of TB, although there was a non-significant decline in bacteriologically confirmed TB cases [All forms of TB (*β* = 0.13; 95% CI: 0.01–0.26; *p* = 0.034), bacteriologically confirmed (*β* = −0.09; 95% CI: −0.21 – 0.02; *p* = 0.100)].

**Table 2:** Comparative ITS analysis model parameters of population-standardized quarterly notification rates of All Forms and bacteriologically-confirmed TB cases for intervention versus control area.

Model parameters	All forms TB		Bacteriologically-confirmed TB	
	β [95% CI]	*p*-value	β [95% CI]	p-value
Baseline rate (β_0_)	14.70 [12.88, 16.52]	<0.001*	11.02 [9.35, 12.68]	<0.001*
Pre-intervention trend, control (*β*1)	0.01 [−0.06, 0.07]	0.822	0.01 [−0.05, 0.07]	0.779
Post-intervention step change, control (β2)	−1.46 [−3.83, 0.90]	0.092	−1.57 [−3.73, 0.60]	0.154
Post-intervention trend, control (β3)	0.04 [−0.05, 0.12]	0.393	0.09 [0.01, 0.17]	0.029*
Difference in baseline (β4)	2.72 [0.15, 5.29]	0.039*	−0.44 [−2.80, 1.92]	0.712
Difference in pre-intervention trends (β5)	−0.09 [−0.18, 0.00]	0.061	−0.02 [−0.10, 0.007]	0.727
Difference in post-intervention step change (β6)	1.39 [−1.95, 4.74]	0.408	2.23 [−0.83, 5.30]	0.150
Difference in post-intervention trends (β7)	0.13 [0.01, 0.26]	0.034*	−0.09 [−0.21, 0.02]	0.100

*Statistically significant at *p* < 0.05.

## Discussion

The study has revealed that engaging private healthcare providers in active TB case-finding strategies can enhance TB case-finding among at-risk urban populations in Sub-Saharan African cities where TB case-finding is declining. By implementing four synchronized TB case detection interventions, we found that community-private healthcare providers detected numerous additional TB cases. The ITS analysis revealed an impact of the intervention on TB case notification rate, though the difference in case notification rate between the intervention and control area was modest. Without the interventions, these cases would have gone undetected or not notified to the NTP, even if they had been detected.

Other studies have also demonstrated that implementing innovative interventions that involve private healthcare providers can enhance TB case detection in urban areas (14–16). In closing the TB case detection gap, some of the innovative PPM interventions implemented in these studies include funding for symptomatic screening, chest x-ray screening, and Xpert MTB/RIF testing, assistance for test logistics, documentation, reporting, and notification to NTP for the private healthcare provider (7, 17, 18). In our study, we efficiently synchronized four interventions with complementary activities to generate demand for TB services among the at-risk group.

The high number of TB screenings in our study was primarily due to the creation of demand for TB services by the various synchronized interventions. Organizations and personnel, including civil society organizations, private healthcare providers, and implementing partners, collaborated to engage community leaders and raise awareness about TB. Private healthcare providers offered TB screening and counseling to most clients, regardless of their initial complaints. Additionally, individuals in close contact with TB patients, such as household members, friends, and co-workers, were followed up and offered TB services and screening. Community screenings were also conducted in our intervention areas.

Unsurprisingly, the community case-finding intervention yielded the most substantial yield of detected TB cases, closely followed by the ICF in private health clinics intervention. This finding is consistent with other findings that highlight the complementarity of successful community TB case finding and ICF in private health clinic interventions (14, 19–21). Effective community engagement projects were found to be complementary to facility-based case finding. To make these interventions more effective, it is important to consider existing linkages within the community and established networks of health providers (22). The importance of community engagement in healthcare and the battle against TB has been shown in other studies as well (23, 24). In our study, all the facilities within the same communities were linked and networked to increase their visibility within the communities. By the end of our intervention, 53 community clinics and hospitals were actively reporting and notifying the NTP of their suspected and confirmed TB cases.

Although the pharmacies and OTCMS intervention identified the highest percentage of presumptive TB cases, it resulted in the lowest proportion of confirmed TB cases, even though all the presumptive cases were tested for TB. This finding is lower compared to other studies (25, 26) and this could be attributed to several reasons. Primarily, the TB screening tool might lack specificity for pharmacies, leading to a high number of false-positive screenings and a significant volume of non-TB respiratory cases (27).

Providing support mechanisms and incentives is crucial for delivering interventions effectively (28). To improve the participation of health workers, we introduced both financial and non-financial incentive approaches. Incentives have always been considered a useful approach to encourage health workers to participate in the PPM program and improve their service performance (29). As part of the program, health workers were given performance-based financial incentives and Trained TB Detectors (TBDs) were introduced in some facilities to assist with TB screening. The introduction of TBDs had a positive impact, particularly in the OTMC intervention, which had previously experienced slow participation.

During the COVID-19 pandemic period, there was an initial decline in TB case notification rates in both the intervention and control areas. The decline in TB case notification rates during the COVID-19 pandemic in Ghana has also been reported by a study by Osei et. al (30), there was a median monthly decline of 21.4% in TB case notifications from April–December, 2020. Likewise, the decline in TB notification has been reported by other studies carried out in Africa (31–34) and beyond (35–38). The underlying reasons for attributing the decline in TB case notification post-intervention to COVID-19 in this study are varied. During the early periods of the pandemic, the government of Ghana implemented various restrictive measures (39). There was the diversion and reprioritizing of health workers, financing, and medical supplies from TB and other control programmes to the COVID-19 response. The NTP had to do a bidirectional screening and testing for TB and COVID-19 among outpatient department attendees during the initial periods of the COVID-19 pandemic (40). The decline in the number of reported cases of TB in the initial quarters of the COVID-19 pandemic might also be due to a reduction in transmission caused by measures such as physical distancing and the use of nose masks. According to the Stop TB Partnership, physical distancing could reduce TB transmission by up to 10% in countries with a high burden of TB (41).

There were a few limitations in our study. The implementation of the interventions faced challenges with insufficient staff for screening in private facilities, high workload for OPD staff, and high staff turnover in private facilities. Before the project began, there was a change in the evaluation area, which reduced the geographic coverage resulting in changes in population sizes. A potential selection bias in the intervention catchment areas may exist, as these areas might have had pre-existing differences in healthcare access or provider engagement that influenced TB case detection. Although we carefully verified each case detected through a data quality audit, we did not collect treatment outcomes because some cases were linked to treatment facilities outside the intervention area. There is the possibility of the Hawthorne effect due to data quality audits and provider incentives to the private facilities and volunteers. We used aggregated data from the NTP for our ITS analysis. However, because this data does not differentiate between public and private sector contributions and also include data outside the intervention period, we were unable to fully determine the reasons for the sharp fluctuations in the graphs outside the intervention period. The COVID-19 pandemic also hindered the implementation of the interventions and the impact of the interventions post-implementation. We could not directly link the decline in TB case notification rates to COVID-19, as this decline could result from several factors. We did not collect data on treatment outcomes because some TB cases were referred to treatment facilities outside the intervention areas, making follow-up and documentation challenging; however, we intend to document treatment outcomes in future studies. Furthermore, there were weak connections between TBDs and diagnosed patients, which complicated effective contact tracing.

This project successfully engaged private healthcare facilities, pharmacies, and OTCMS in active case-finding for TB within urban slum communities, leading to an increase in TB case detection. Notably, some private healthcare facilities have continued to offer free TB screening services even after the project’s implementation period, indicating the potential for sustainability of this model. To ensure long-term sustainability and scalability, these interventions should be integrated into national and local TB control strategies. This can be achieved by expanding the PPM DOT model by the NTP, securing stable funding from both domestic and international sources, and enhancing digital reporting systems for real-time data tracking. Strengthening electronic data capture will improve patient tracking and enable prompt reimbursement through the NHIS, which should be expanded to cover TB services provided by private healthcare providers. These strategic policy adjustments will help maintain the progress made and facilitate the replication of successful models in similar urban settings facing declining TB detection rates.

## Conclusion

By extending the free TB service to private healthcare providers and maintaining continuous community engagement, it is possible to increase case notification in vulnerable urban populations where TB case finding is declining. It is beneficial to utilize and strengthen the networks between private and public actors in the health sector to tackle diseases of public health importance. For countries that aim to reactivate their dormant PPM programs or are in the process of developing strategies for PPM as a national program, our study can provide valuable insights and guidance for future directions.

## Data Availability

The original contributions presented in the study are included in the article/Supplementary material, further inquiries can be directed to the corresponding author.
