# Supplementary material for: Scaling up tuberculosis case finding via private providers in Ghana: an impact evaluation using interrupted time series
Source: Front Public Health. 2025 Aug 26;13:1598269. doi: 10.3389/fpubh.2025.1598269 (PMC12417460; doi:10.3389/fpubh.2025.1598269)
Supplement: Supplementary file 1 [file Supplementary_file_1.docx]

**Supplementary Information**

**Screening criteria and eligibility for sputum collection and examination with GeneXpert test.**

**A. Facility and pharmacy-based screening (excluding People Living with HIV)**

Cough >2 weeks with or without additional TB symptom

Cough <2 weeks with at least one other additional TB symptom

Abnormal chest X-ray with or without TB symptom

**B. People Living with HIV**

Any one or more TB symptoms

Abnormal Chest X-ray with or without symptom

**C. Contact tracing and Community-Based Screening**

Abnormal Chest X-ray with or without TB symptoms

Any one or more of TB symptoms

**Any signs and symptoms for TB include cough >2 weeks or a cough of any duration with at least one of other symptoms; chest pain fever, unintentional weight loss, and night sweats*

**Description of regression model and model parameters used for**

The parameters were obtained for a segmented regression model with the following structure:

Yt = β_0_ + β_1_Tt + β_2_Xt + β_3_XtTt + β_4_Z + β_5_ZTt + β_6_ZXt + β_7_ZXtTt + ϵt.

| Yt | case notification rate per quarter at quarter t |
| --- | --- |
| Tt | number of quarters since the start of analysis |
| Xt | 0 if t prior to intervention, 1 if t during intervention |
| Z | 0 if Control area, 1 if Intervention area (intervention cohort) |
| εt | Variability at time t |

ZTt, ZXt, and ZXtTt are interaction terms where T restarts at 0 at the start of the intervention (Q4 2018)

The exponentiated regression coefficients (β0 to β7) represent case notification rate ratios (RR) for every unit increase in time (quarters). The coefficients β0 to β3 represent the control group as follows: β0, intercept; β1, pre-intervention trend in the control group; β2, intervention level change in control group in first quarter of intervention (post-intervention step change); β3, trend in control grou during intervention (post-intervention trend). β4 to β7 represent differences between the control and intervention districts: β4, difference in baseline intercepts between the intervention and control groups; β5, difference in preintervention trends; β6, difference in intervention level change at first quarter of intervention between intervention and control groups; β7, difference in the trend between intervention and control groups during the intervention.

References:

1. Linden, A. A comprehensive set of post estimation measures to enrich interrupted time-series analysis. Stata J. 2017, 73–88, doi:10.1177/1536867X1701700105.

2. Linden, A. Conducting interrupted time-series analysis for single- and multiple-group comparisons. Stata J. 2015, 15, 480–500, doi:10.1177/1536867X1501500208.

**Supplementary Table (S1): Quarterly all-forms TB and bacteriological-confirmed case notifications rates from Q1 2015 to Q4 2022, for the intervention area and control area**

|  | **2015** | | | | **2016** | | | | **2017** | | | | **2018** | | | |
| --- | --- | --- | --- | --- | --- | --- | --- | --- | --- | --- | --- | --- | --- | --- | --- | --- |
|  | Q1 | Q2 | Q3 | Q4 | Q1 | Q2 | Q3 | Q4 | Q1 | Q2 | Q3 | Q4 | Q1 | Q2 | Q3 | Q4 |
| **Notification (All forms of TB)** | | | | | | | | | | | | | | | | |
| Intervention | 451 | 399 | 386 | 391 | 461 | 395 | 353 | 363 | 389 | 383 | 340 | 328 | 392 | 385 | 300 | 315 |
| Control | 200 | 219 | 200 | 230 | 242 | 220 | 183 | 199 | 222 | 212 | 214 | 201 | 242 | 224 | 204 | 183 |
| **Notification (Bacteriological confirmed TB cases)** | | | | | | | | | | | | | | | | |
| Intervention | 276 | 255 | 239 | 270 | 289 | 244 | 234 | 262 | 260 | 256 | 233 | 247 | 274 | 286 | 235 | 250 |
| Control | 155 | 168 | 143 | 174 | 182 | 164 | 129 | 157 | 166 | 146 | 176 | 163 | 174 | 179 | 147 | 128 |
| **Notification rate (All forms TB cases per 100,000)** | | | | | | | | | | | | | | | | |
| Intervention | 18.3 | 16.2 | 15.6 | 15.8 | 18.7 | 16.0 | 14.3 | 14.7 | 15.8 | 15.5 | 13.8 | 13.3 | 15.9 | 15.6 | 12.1 | 12.8 |
| Control | 13.9 | 15.2 | 13.9 | 16.0 | 16.8 | 15.3 | 12.7 | 13.8 | 15.4 | 14.7 | 14.9 | 14.0 | 16.8 | 15.6 | 14.2 | 12.7 |
| **Notification rate (Bacteriological confirmed TB cases per 100,000)** | | | | | | | | | | | | | | | | |
| Intervention | 11.2 | 10.3 | 9.7 | 10.9 | 11.7 | 9.9 | 9.5 | 10.6 | 10.5 | 10.4 | 9.4 | 10.0 | 11.1 | 11.6 | 9.5 | 10.1 |
| Control | 10.8 | 11.7 | 9.9 | 12.1 | 12.6 | 11.4 | 9.0 | 10.9 | 11.5 | 10.1 | 12.2 | 11.3 | 12.1 | 12.4 | 10.2 | 8.9 |
|  |  |  |  |  |  |  |  |  |  |  |  |  |  |  |  |  |
|  | **2019** | | | | **2020** | | | | **2021** | | | | **2022** | | | |
|  | Q1 | Q2 | Q3 | Q4 | Q1 | Q2 | Q3 | Q4 | Q1 | Q2 | Q3 | Q4 | Q1 | Q2 | Q3 | Q4 |
| **Notification (All forms of TB)** | | | | | | | | | | | | | | | | |
|  | 356 | 373 | 392 | 341 | 503 | 321 | 403 | 375 | 371 | 401 | 369 | 357 | 439 | 445 | 426 | 528 |
|  | 168 | 192 | 218 | 200 | 252 | 182 | 259 | 227 | 222 | 242 | 186 | 201 | 192 | 235 | 252 | 206 |
| **Notification (Bacteriological confirmed TB cases)** | | | | | | | | | | | | | | | | |
|  | 321 | 307 | 305 | 234 | 296 | 195 | 270 | 241 | 234 | 225 | 214 | 229 | 273 | 292 | 279 | 306 |
|  | 131 | 160 | 167 | 169 | 194 | 143 | 211 | 190 | 174 | 204 | 149 | 165 | 283 | 181 | 209 | 195 |
| **Notification rate (All forms TB cases per 100,000)** | | | | | | | | | | | | | | | | |
|  | 14.4 | 15.1 | 15.9 | 13.8 | 20.4 | 13.0 | 16.3 | 15.2 | 15.0 | 16.2 | 14.9 | 14.5 | 17.8 | 18.0 | 17.3 | 21.4 |
|  | 11.7 | 13.3 | 15.1 | 13.9 | 17.5 | 12.6 | 18.0 | 15.8 | 15.4 | 16.8 | 12.9 | 14.0 | 13.3 | 16.3 | 17.5 | 14.3 |
| **Notification rate (Bacteriological confirmed TB cases per 100,000)** | | | | | | | | | | | | | | | | |
|  | 13.0 | 12.4 | 12.4 | 9.5 | 12.0 | 7.9 | 10.9 | 9.8 | 9.5 | 9.1 | 8.7 | 9.3 | 11.1 | 11.8 | 11.3 | 12.4 |
|  | 9.1 | 11.1 | 11.6 | 11.7 | 13.5 | 9.9 | 14.7 | 13.2 | 12.1 | 14.2 | 10.4 | 11.5 | 19.7 | 12.6 | 14.5 | 13.5 |

**Regression analysis of all forms of TB cases in the intervention and control areas from 2015 to 2022, with a pink line representing the counterfactual in the absence of intervention.**

**Regression analysis of bacteriologically confirmed TB cases in the intervention and control areas from 2015 to 2022, with a pink line representing the counterfactual in the absence of intervention.**
